# Epigenetic silencing of the 3p22 tumor suppressor *DLEC1* by promoter CpG methylation in non-Hodgkin and Hodgkin lymphomas

**DOI:** 10.1186/1479-5876-10-209

**Published:** 2012-10-11

**Authors:** Zhaohui Wang, Lili Li, Xianwei Su, Zifen Gao, Gopesh Srivastava, Paul G Murray, Richard Ambinder, Qian Tao

**Affiliations:** 1 Shenzhen Institutes of Advanced Technology (SIAT), Chinese Academy of Sciences (CAS)-CUHK, Shenzhen, China; 2 Cancer Epigenetics Laboratory, Department of Clinical Oncology, State Key Laboratory of Oncology in South China, Sir YK Pao Center for Cancer, The Chinese University of Hong Kong and CUHK Shenzhen Research Institute, Shatin, Hong Kong; 3 Department of Pathology, Peking University Health Science Center, Beijing, China; 4 Department of Pathology, University of Hong Kong, Shatin, Hong Kong; 5 Cancer Research UK Institute for Cancer Studies, University of Birmingham, Birmingham, UK; 6 Johns Hopkins Singapore and Sidney Kimmel Comprehensive Cancer Center, Johns Hopkins School of Medicine, Baltimore, MD, USA

**Keywords:** DLEC1, CpG, Methylation, Tumor suppressor, Lymphoma

## Abstract

**Background:**

Inactivaion of tumor suppressor genes (TSGs) by promoter CpG methylation frequently occurs in tumorigenesis, even in the early stages, contributing to the initiation and progression of human cancers. *Deleted in lung and esophageal cancer 1* (*DLEC1*), located at the 3p22-21.3 TSG cluster, has been identified frequently silenced by promoter CpG methylation in multiple carcinomas, however, no study has been performed for lymphomas yet.

**Methods:**

We examined the expression of *DLEC1* by semi-quantitative reverse transcription (RT)-PCR, and evaluated the promoter methylation of *DLEC1* by methylation-specific PCR (MSP) and bisulfite genomic sequencing (BGS) in common lymphoma cell lines and tumors.

**Results:**

Here we report that *DLEC1* is readily expressed in normal lymphoid tissues including lymph nodes and PBMCs, but reduced or silenced in 70% (16/23) of non-Hodgkin and Hodgkin lymphoma cell lines, including 2/6 diffuse large B-cell (DLBCL), 1/2 peripheral T cell lymphomas, 5/5 Burkitt, 6/7 Hodgkin and 2/3 nasal killer (NK)/T-cell lymphoma cell lines. Promoter CpG methylation was frequently detected in 80% (20/25) of lymphoma cell lines and correlated with *DLEC1* downregulation/silencing. Pharmacologic demethylation reversed *DLEC1* expression in lymphoma cell lines along with concomitant promoter demethylation. *DLEC1* methylation was also frequently detected in 32 out of 58 (55%) different types of lymphoma tissues, but not in normal lymph nodes. Furthermore, *DLEC1* was specifically methylated in the sera of 3/13 (23%) Hodgkin lymphoma patients.

**Conclusions:**

Thus, methylation-mediated silencing of *DLEC1* plays an important role in multiple lymphomagenesis, and may serve as a non-invasive tumor marker for lymphoma diagnosis.

## Introduction

Epigenetic silencing of tumor suppressor genes (TSGs) by promoter CpG methylation and histone modification has been widely recognized as one of the major causes of tumorigenesis including hematological malignancies [1,2]. Aberrant methylation of TSG is frequently detected even in the early stage of tumorigenesis, suggesting its potential as tumor biomarker for early detection and therapeutic targeting.

Deletions of the 3p22-21.3 region have been identified as one of the earliest molecular events in various malignancies [3], including naspharyngeal [4], head and neck [5], lung [6], gastric [7], breast [8], cervix [9] and renal [10] carcinomas, as well as lymphomas [11]. A growing number of TSGs, including *RASSF1A*[12,13], *BLU*/*ZMYND10*[14,15], and *CACNA2D2*[16], have been identified in this region [17,18]. Frequent inactivation of several 3p21.3 genes as functional TSGs, such as *RASSF1* and *BLU*[14,19], by promoter CpG methylation had been identified associated with tumor initiation and progression. For example, promoter methylation of *RASSF1A* has been shown related to poor prognosis and advanced tumor stage of certain tumor types [20-33]. Restoration of RASSF1A in cancer cell lines inhibited tumor cell growth and metastasis [34]. Thus, 3p22-21.3 is a critical TSG cluster in tumorigenesis [3,18].

*Deleted in lung and esophageal cancer 1* (*DLEC1*), a 3p22 cluster genes, was first identified as a TSG in esophageal and lung cancers [35]. Downregulation of *DLEC1* by promoter methylation has been found in multiple cancers, including nasopharyngeal [36,37], ovarian [38], lung [39], hepatocellular [40], gastric [41], renal [42], and breast carcinomas [43], suggesting its potential as a broad TSG [44,45]. Remarkably, *DLEC1* was methylated in breast cancer as well as pre-invasive lesions but rarely in normal breast tissues, indicating its potential as an epigenetic marker for early tumors [43].

In this study, we examined the expression and methylation status of *DLEC1* in lymphoma cell lines and tissues, and evaluated its potential as a tumor marker for the early detection of hematologic tumors.

## Methods

### Cell lines and tumor samples

Non-Hodgkin and Hodgkin lymphoma cell lines studied included diffuse large B-cell lymphoma (DLBCL) cell lines (OCI-Ly1, Ly3, Ly7, Ly8, Ly18, SUDHL6); peripheral T cell lymphoma (PTCL) cell lines (Ly13.2, Ly17); Burkitt lymphoma (BL) cell lines (AG876, BJAB, Namalwa, Rael, Raji); Hodgkin lymphoma (HL) cell lines (L428, L540, L591, L1236, KM-H2, HD-LM-2, HD-MY-Z) (DSMZ cell collection, Braunschweig, Germany); and natural killer (NK)/T-cell lines (NL) (KHYG-1, SNK6, YT) [46-48]. Cell lines were maintained in RPMI1640 or Dulbecco’s Modified Eagle’s Medium supplemented with 10% fetal calf serum (FBS) (Invitrogen, Paisley, Scotland) and 1% streptomycin/penicillin at 37°C in 5% CO_2_. HL cell lines were treated with 5 μM of 5-aza-2^′^-deoxycytidine (Aza) (Sigma) for 3 days, or further treated with 100 nmol/L trichostatin A (Cayman Chemical Co., Ann Arbor, MI, USA) for additional ~16 h as described previously [15,49], and L428 and KM-H2 cell lines were treated with Aza for 6 days.

Normal peripheral blood mononuclear cells (PBMC), lymph node samples, different types of lymphoma samples, as well as sera from healthy individuals and HL patients were collected as previously described, and the reliability and quality of all the studied samples in this study have been confirmed before [41,47,50]. The study was approved by Johns Hopkins Medicine Institutional Review Board. Normal adult tissue RNA samples were purchased commercially (Stratagene, La Jolla, CA, USA or Millipore-Chemicon, Billerica, MA, USA). DNA and RNA were extracted from lymphoma cell lines and primary lymphomas using TRIzol reagent (Invitrogen) as previously described [51,52].

### Semi-quantitative reverse transcription (RT)-PCR

RT-PCR was performed as previously described [40,41]. Primers used for RT-PCR are: *DLEC1*A: 5^′^-ttcctccctcgcctactc, *DLEC1*B: 5^′^-aaactcatccagccgctg; GAPDH33: 5^′^-gatgaccttgcccacagcct, GAPDH55: 5^′^-atctctgccccctctgctga. RT-PCR was done with 32 cycles for *DLEC1* and 23 cycles for GAPDH.

### Bisulfite treatment and promoter methylation analysis

Bisulfite modification of DNA, methylation-specific PCR (MSP) and bisulfite genomic sequencing (BGS) were performed as described [53,54]. MSP primers for *DLEC1* were: *DLEC1*m1: 5^′^-gtttcgtagttcggtttcgtc; *DLEC1*m2: 5^′^-cgaaatatcttaaatacgcaacg; *DLEC1*u1: 5^′^-tagttttgtagtttggttttgtt; *DLEC1*u2: 5^′^-acaaaatatcttaaatacacaaca. For BGS, bisulfite-treated DNA was amplified using primers *DLEC1*BGS1: 5^′^-gaagatataaatgtttataatgatt; *DLEC1*BGS4: 5^′^-aactacaaccccaaatcctaa. *ANKRD30A*m1: 5^′^-cggtagttgttatttgtacgc; *ANKRD30A*m2: 5^′^-tcctctctcaataaaatcgcg. MSP and BGS were performed for 40 cycles by using the AmpliTaq-Gold DNA polymerase (Applied Biosystems). All primer sets were previously tested for not amplifying any unbisulfited DNA. Amplified BGS products were cloned and 5 to 10 colonies were randomly chosen for sequencing.

## Results

### *DLEC1* was downregulated by promoter CpG methylation in lymphoma cell lines

**Figure 1:**
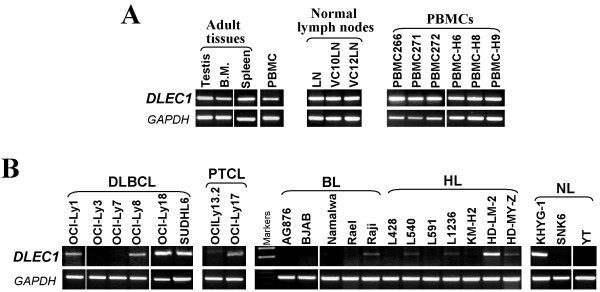
***DLEC1 *is downregulated in lymphoma cell lines.A.** Expression analysis of *DLEC1* in normal adult tissues, lymph nodes, and PBMC samples. **B.** Representative analyses of *DLEC1* expression by RT-PCR in lymphoma cell lines. *GAPDH* was used as internal control. B.M: bone marrow; DLBCL: diffuse large B-cell lymphoma; PTCL: peripheral T cell lymphoma; BL: Burkitt lymphoma; HL: Hodgkin lymphoma; NL: nasal NK/T-cell lymphoma.

We first examined the expression of *DLEC1* mRNA in 25 non-Hodgkin and Hodgkin lymphoma cell lines by RT-PCR. *DLEC1* was highly expressed in the normal lymph node, PBMC samples, as well as human adult testis and bone marrow tissues (Figure 1A), but silenced or reduced in 33% (2/6) DLBCL, 50% (1/2) PTCL, 100% (5/5) BL, 86% (6/7) HL, and 67% (2/3) NL cell lines (Figure 1B), indicating that *DLEC1* is a candidate TSG for lymphomas.

**Figure 2:**
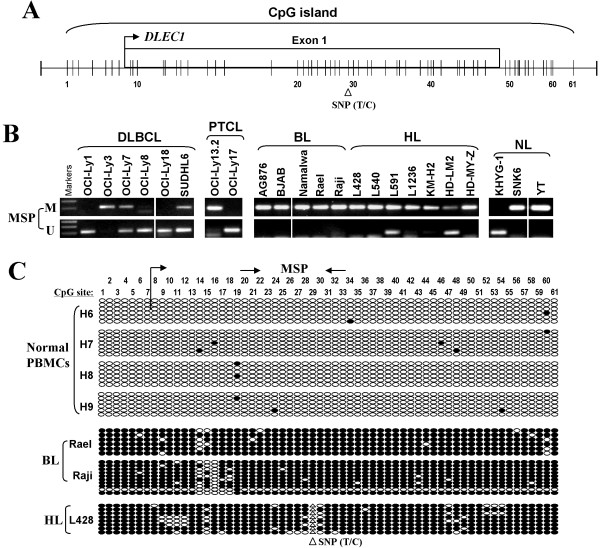
**Promoter CpG methylation of *DLEC1 *in lymphoma cell lines.A.** Diagram shows the *DLEC1* CpG island, exon 1 (indicated with a rectangle), and CpG sites (short vertical lines). Numbers from 1 to 61 indicate the individual CpG sites. **B.***DLEC1* methylation as measured by MSP in lymphoma cell lines. M: methylated; U: unmethylated; DLBCL: diffuse large B-cell lymphoma; BL: Burkitt lymphoma; HL: Hodgkin lymphoma; NL: nasal NK/T-cell lymphoma. **C.** High resolution mapping of the methylation status of the *DLEC1* promoter by BGS in normal PBMCs, and representative BL and HL cell lines. The *DLEC1* transcription start site is shown as bent arrows. Circles, CpG sites analyzed; row of circles, an individual promoter allele that was cloned, randomly selected, and sequenced; filled circle, methylated CpG site; open circle, unmethylated CpG site.

The promoter and exon 1 region of *DLEC1* is a typical CpG island, with a T/C SNP site at CpG#29 (Figure 2A). MSP analysis showed that *DLEC1* promoter methylation was frequently detected in 3/6 DLBCL, 1/2 PTCL, 5/5 BL, 7/7 (one weak) HL, and 2/3 NL cell lines (Figure 2B; Table 1), well correlated with its silencing or reduction. *DLEC1* methylation was further verified in detail by BGS analysis of 61 CpG sites within the CpG island. CpG sites of *DLEC1* examined were heavily methylated in BL cell lines Rael, Raji, and in the HL cell line L428, but rarely in all normal PBMC samples, which confirmed the MSP data (Figure 2C). These results indicate that *DLEC1* silencing by promoter methylation is a critical event in lymphomagenesis.

**Table 1:** **Frequencies of *DLEC1 *methylation in various lymphoma tissues**

	**Lymphoma**	***DLEC1 *promoter (methylated)**
Cell lines	Diffuse large B‑cell lymphoma	50% (3/6)
	Peripheral T-cell lymphoma	50% (1/2)
	Burkitt lymphoma	100% (5/5)
	Hodgkin lymphoma	100% (7/7, one weak)
	Nasal NK/T-cell lymphoma	67% (2/3)
Tumors	Burkitt lymphoma	83% (5/6)
	Diffuse large B‑cell lymphoma	10% (1/10)
	Nasal NK/T‑cell lymphoma	75% (6/8)
	Hodgkin lymphoma	53% (16/30, one weak)
	Follicular lymphoma	100% (4/4, two weak)
	HL sera	23% (3/13)
Normal samples	Normal lymph node	0/9
	Normal sera	0/20

### Silencing of *DLEC1* could be reversed by pharmacologic demethylation

**Figure 3:**
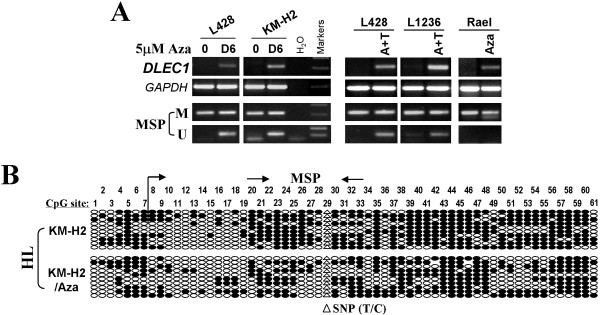
**Pharmacologic demethylation restored *DLEC1 *expression in methylated/silenced lymphoma cell lines.A.** Expression and methylation analyses of *DLEC1* in HL cell lines with Aza treatment or combined with TSA (A+T). M: methylated; U: unmethylated. **B.** BGS analysis of *DLEC1* methylation in lymphoma cell line KM-H2 before and after 5 μM Aza treatment.

To further confirm whether promoter methylation mediates the loss of *DLEC1* expression in lymphoma, lymphoma cell lines with methylated and reduced *DLEC1* were treated with the DNA methylation inhibitor, Aza, alone or combined with the HDAC inhibitor trichostatin A (TSA). After Aza treatment, *DLEC1* expression was significantly induced in cell lines Rael, L428, L1236 and KM-H2. MSP analysis showed increased unmethylated alleles. Similar results were obtained using a combination treatment of Aza and TSA (Figure 3). Demethylation of the *DLEC1* promoter was further confirmed by BGS. These results confirmed that promoter CpG methylation directly mediates *DLEC1* silencing in lymphomas.

### *DLEC1* is frequently methylated in primary lymphomas

**Figure 4:**
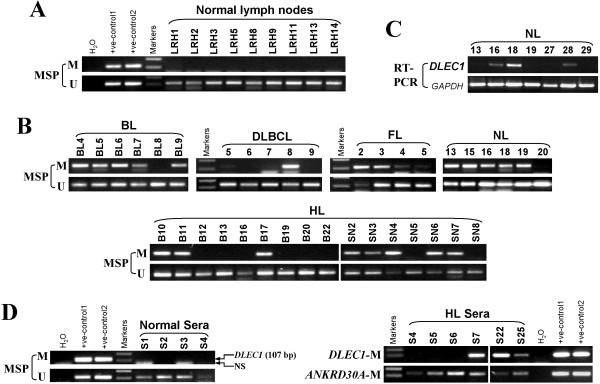
***DLEC1 *is frequently methylated in lymphoma tissues. A.** MSP analysis of *DLEC1* methylation in normal lymph node samples. Templates used for positive controls are: bisulfite treated DNA from HCT116 (+ve-control1) and HT-29 (+ve-control2) cell lines for methylation; SW480 (+ve-control1) and SNU16 (+ve-control2) cell lines for unmethylation. **B.** Representative analyses of *DLEC1* methylation in primary lymphomas by MSP in primary lymphoma BL, DLBCL, HL and FL samples. BL: Burkitt lymphoma; DLBCL: diffuse large B-cell lymphoma; HL: Hodgkin lymphoma; NL: nasal NK/T-cell lymphoma; FL: follicular lymphoma. **C.** Representative analysis of *DLEC1* expression in NL tumors. *GAPDH* was used as an internal control. **D.** Representative MSP analysis of *DLEC1* methylation in serum samples from HL patients and normal controls. Positive controls: bisulfite treated DNA from HCT116 (+ve-control1) and HT-29 (+ve-control2) cell lines. M: methylated; NS: non-specific band.

We next investigated *DLEC1* methylation in different types of primary lymphomas. Of 58 lymphoma tissues, *DLEC1* methylation was detected in 5/6 (83%) BL, 16/30 (53%, one weak) HL, 1/10 (10%) DLBCL, 6/8 (75%) NL, and 4/4 (100%, two weak) follicular lymphoma (FL) tumor samples, while no methylation was detected in normal lymph node samples (Figure 4A and B; Table 1). Unmethylated bands were detected in all samples due to the inevitable inclusion of non-tumor cells in the analysis. Furthermore, the reduction or silencing of *DLEC1* expression was observed in 6 out of 7 (86%) NL tumors as measured by RT-PCR.

Furthermore as a pilot study, we examined *DLEC1* methylation in serum samples of 13 HL patients with sera from 20 healthy individuals as controls. *ANKRD30A* is a normally methylated gene, thus used as an internal control for validating genomic DNA integrity of these samples, in addition to unmethylated *DLEC1*. Results showed that *DLEC1* methylation was detected in 3/13 (23%) sera from HL patients (Figure 4D) but not in any normal sera.

## Discussion

*DLEC1* is located in the commonly deleted region 3p22-21.3 [3,18]. A cluster of TSGs, including *RASSF1A*, *BLU*, and *CACNA2D2*, has been identified within this region. Silencing of *RASSF1A*, *BLU*, as well as *DLEC1* by promoter CpG methylation had been extensively identified in multiple human cancers. Notably, promoter methylation of several 3p22-21.3 TSGs, such as *RASSF1A* and *BLU*, has also been identified in lymphomas [50,55], suggesting that TSGs in this region are frequently susceptible to epigenetic disruption during lymphoma pathogenesis.

Here, we report that *DLEC1* is frequently silenced by promoter methylation both in lymphoma cell lines and primary lymphomas, but seldom in normal lymph nodes and PBMCs. Pharmacologic demethylation could reactivate *DLEC1* expression, suggesting that epigenetic mechanism including DNA methylation and histone modification mediates *DLEC1* transcriptional silencing. As primary tumor tissues usually contain infiltrating non-malignant cells such as lymphocytes, unmethylated alleles were detected in all the tumor samples which also acting as an internal control for genomic DNA integrity. *DLEC1* unmethylated alleles and *ANKRD30A* methylated alleles detected in different types of normal and lymphoma samples indicated the reliability of these samples as shown before. *DLEC1* methylation showed a relatively low frequency in DLBCL samples, compared to other lymphoma types. The reason for this difference is not clear probably due to the special biologic features of DLBCL, which needs to be further confirmed by large sample size. Our results suggest that epigenetic silencing of *DLEC1* is important for lymphoma pathogenesis. Remarkably, *DLEC1* is specifically methylated in sera of HL patients, suggesting its potential as an epigenetic biomarker for the non-invasive diagnosis of lymphomas.

## Conclusions

This study identifies the frequent epigenetic inactivation of *DLEC1* in various lymphomas and demonstrates its potential as a non-invasive tumor marker for the detection of lymphomas. More molecular studies on the tumor suppressive functions of *DLEC1* in lymphoma pathogenesis are needed.

## Competing interests

The authors declare no conflict of interest.

## Authors’ contributions

ZW and LL analyzed data and drafted the manuscript. XS and ZW acquired data. ZG, GS, PGM and AR provided material and reviewed the manuscript. PGM contributed to the writing of the manuscript. QT conceived and supervised the study, analyzed data and finalized the manuscript. All authors read and approved the final manuscript.
